# Transient Receptor Potential Channels in Prostate Cancer: Associations with ERG Fusions and Survival

**DOI:** 10.3390/ijms26083639

**Published:** 2025-04-11

**Authors:** Nirosha J. Murugan, Emma Genautis, Ioannis A. Voutsadakis

**Affiliations:** 1 Department of Biology, Wilfrid Laurier University, Waterloo, ON N2L 6C2, Canada; 2 Allen Discovery Center, Tufts University, Medford, MA 02155, USA; 3 Algoma District Cancer Program, Sault Area Hospital, Sault Ste. Marie, ON P6B 0A8, Canada; 4 Section of Internal Medicine, Division of Clinical Sciences, Northern Ontario School of Medicine, Sudbury, ON P3E 2C6, Canada

**Keywords:** calcium, signaling, biomarker, prognosis, cation regulation, transient receptor potential

## Abstract

Calcium movement and concentration in the cell plays significant roles in normal physiology and in diseases such as cancer. The significance of this ion in oncogenesis suggests that membrane-relevant proteins are involved in its regulation and are deregulated in various cancers. These channels and transporters could be targets for therapeutic interventions. An evaluation of the expression of transient receptor potential (TRP) channels in prostate cancer was performed using publicly available genomic and proteome data. Two TRP family members with high expression in prostate cancers, TRPML2 and TRPM4, were chosen for further analysis the uncover the associations of their level of expression with clinical and pathologic prostate cancer characteristics. Several TRP channels were expressed in prostate cancers at the protein level including TRPM4, TRPML1, TRPML2, TRPC1 and TRPP3. At the mRNA level, MCOLN2 and TRPM4 were strongly expressed in a sub-set of prostate cancers. Cases with high MCOLN2 mRNA expression were associated with frequent ERG fusions and a trend for better survival outcomes. In contrast, prostate cancer cases with high TRPM4 mRNA expression were associated with lower ERG fusion frequency than cases with low TRPM4 mRNA expression. The prognosis of prostate cancers with high TRPM4 expression was not different from the prognosis with counterparts having low TRPM4 mRNA expression. TRP channels were expressed in sub-sets of prostate cancers. The two well-expressed channels of the super family, TRPML2 and TRPM4, have divergent associations with the most prevalent prostate cancer molecular aberrations, ERG fusions. These results imply diverse regulations of the TRP channels that would have to be taken into consideration when devising therapeutic interventions targeting individual channels.

## 1. Introduction

Prostate cancer is the most prevalent type of cancer in men, with an estimated 300,000 new cases diagnosed in the United States of America in 2024 [1]. This represents about 30% of all cancer diagnoses in men for the same year. In Canada, prostate cancer is also the most prevalent cancer in men. Effective therapies including hormonal therapies and chemotherapy have increased survival for both castration-sensitive and castration-resistant disease [2]. Despite these improvements, the disease continues to be lethal in a significant proportion of patients. with an estimated 35,000 deaths in the United States of America in 2024 [1]. Therefore, novel therapies are still urgently needed. The development of new drugs and the identification of novel targets for drug repurposing can be aided by the improved understanding of the molecular pathophysiology of the disease. ERG (ETS-Related Gene) is one of the 28 transcription factors of the ETS (E26 Transformation-Specific) family and is involved in fusions with the Androgen Receptor (AR)-regulated gene TMPRSS2 (Transmembrane Serine Protease 2) in 40% to 70% of prostate cases in different series [3,4,5]. These fusions result in the up-regulation of ERG expression under AR transcriptional regulation.

Cancer cells have higher membrane impedance, which makes them less electrically conductive than normal cells [6]. This can affect the flow of electrical signals within the cell, leading to changes in cellular behavior, such as increased proliferation, reduced apoptosis and enhanced invasion and migration capabilities. Emerging therapeutic approaches are based on the idea that electric fields can be used to disrupt the behavior of cancer cells by affecting the activity of ion channels to induce selective apoptosis [7]. Complementary approaches based on bioelectricity have also been considered to enhance the effectiveness of existing therapeutic strategies [8].

Differences in charge across cell membranes establish the cell’s membrane potential, termed as the bioelectrical activity of the cell [9,10]. Membrane bioelectrical activity is well-studied in the context of the firing and proliferation of action potentials in excitable cells such as neurons. However, membrane potentials are likewise a useful bioelectric signal in non-excitable cells, such as prostate tissue [11]. Membrane potentials have been revealed to be, on average, more depolarized in cancer cells compared to respective benign cells of origin, making this a useful indicator of malignancy [6]. The detection of differences in membrane potential between malignant and benign cells represents a promising method for cancer diagnosis [12,13,14].

Physiologically, the regulation of calcium levels in the cell cytoplasm is performed by diverse calcium channels, transporters and receptors located in the cell membrane or the membranes of the intracellular organelles, including the endoplasmic reticulum, mitochondria and lysosomes [15,16,17,18,19,20]. These receptors belong to several families that include voltage-dependent ion channels (VDAC), voltage-gated calcium channels (VGCCs), Orai family channels and transient receptor potential (TRP) channels [21]. In addition to its impact on membrane potential, calcium serves as a potent second messenger, transducing and amplifying signals for the activation of protein cascades downstream, affecting a variety of cell functions [22,23].

TRP channels constitute a family of membrane channels that sense various environmental or intracellular signals according to their location and facilitate the transport of ions; these ions are, most prominently, calcium, but also sodium and potassium [24]. TRP channels are conserved across eukaryotes, conferring several types of transduction beyond chemoreception, including biophysical modalities such as mechanoreception and photoreception. TRPs are categorized in several sub-families that include the canonical family with six members (TRPC1 to TRPC6), the ankyrin family with a single member, TRPA1, the melastatin family with eight members (TRPM1 to TRPM8), the vanilloid family with six members (TRPV1 to TRPV6), the mucolipid family with three members (TRPML1 to TRPML3, encoded by genes *MCOLN1* to *MCOLN3*, respectively) and the polycystin family, also with three members (TRPP1 to TRPP3 encoded by genes *PKD2*, *PKD2L1* and *PKD2L2*, respectively) [25]. TRP channels present a conserved structure of six transmembrane domains flanked by an aminoterminal domain and a carboxyterminal domain, both located in the inside of the cell. The pore allowing cation passage is located between transmembrane domains 5 and 6. The aminoterminal and carboxyterminal domains of TRP channels differ between members of the different TRP families and provide the specificity for regulatory and activator interactions. TRP members may be activated by physical stimuli such as temperature, light and pressure or chemical stimuli including lipids, reactive oxygen species and an array of exogenous chemicals, such as allicin, acrolein, capsaicin and parthenolide [25,26]. The deregulation of TRPs and other ion channels function during cancer development, leading to cancer-promoting but also anti-carcinogenic effects, is well-described [27]. However, it is not clear to what degree channel dysfunction is a result of the widespread deregulation of physiological processes in cancer cells or has a causative effect in propagating cancer development and progression.

This research examines the expression, alterations and prognostic implications of TRPs in prostate cancer, taking advantage of published genomic and proteomic studies.

## 2. Results

**Figure 1:**
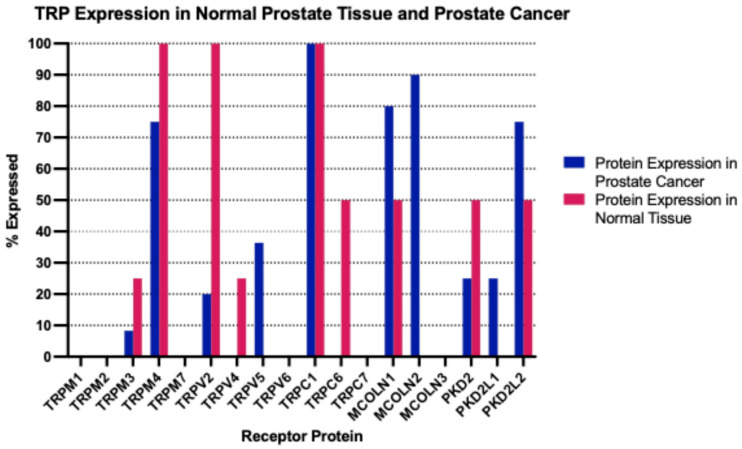
Percentage of expression of TRP channels by immunohistochemistry in normal prostate samples and samples from prostate cancer. The percentage number refers to the percentage of histologic section samples of normal prostate and of prostate cancer that stain positive for the respective TRP channels. Data are from the Human Protein Atlas.

The mRNA expression of TRP channels in normal prostate tissues varied significantly. Several TRP channels were not expressed (TRPM1, TRPV5, TRPC5, TRPC7) or were expressed in low levels (<1 nTPM (normalized Transcripts per Million): TRPA1, TRPM2, TRPM3, TRPM5, TRPM6, TRPV3, TRPC3, MCOLN2, MCOLN3, PKD2L1, PKD2L2). Other TRP channels were expressed in intermediate levels (1–10 nTPM: TRPV1, TRPV2, TRPV4, TRPC1, TRPC4, TRPC6, MCOLN1) or high levels (>10 nTPM: TRPM4, TRPM7, TRPM8, TRPV6, PKD2). At the protein level, the human protein atlas had evaluated the expression of several TRP channels in normal prostate tissues and found consistently moderate to high expression in all samples examined for TRPM4, TRPV2 and TRPC1 (Figure 1).

The genes encoding for TRP channels were rarely mutated in prostate cancer. In the prostate cancer cohort of TCGA, that included 494 patients, all genes encoding for TRP channels were mutated in 0 to 6 cases (0 to 1.2%). The most frequently mutated gene was *TRPM6* with six mutated cases (1.2%) in the cohort. Copy number alterations were also observed rarely with the exception of the amplification of TRPA1, which was amplified in 7.2% of prostate cancers in TCGA, and of TRPC4 and TRPC3, which were homodeleted in 5.9% and 2.5% of cases, respectively. Similarly, in the MSK cohort, TRPA1 was amplified in 6.3% of prostate cancers and TRPC4 and TRPC3 were homodeleted in 4.4% and 1.5% of cases, respectively.

**Figure 2:**
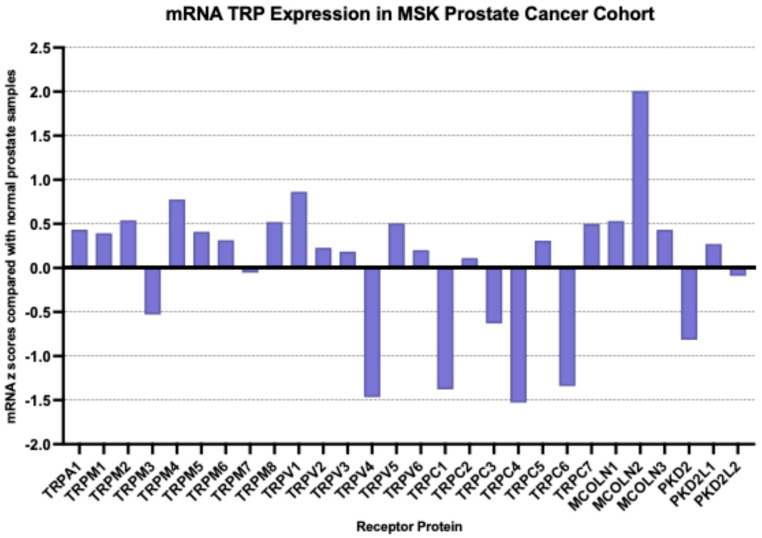
mRNA expression of TRP channels in prostate cancer (mRNA z scores compared with normal samples). Data are from the MSK prostate cancer cohort.

**Figure 3:**
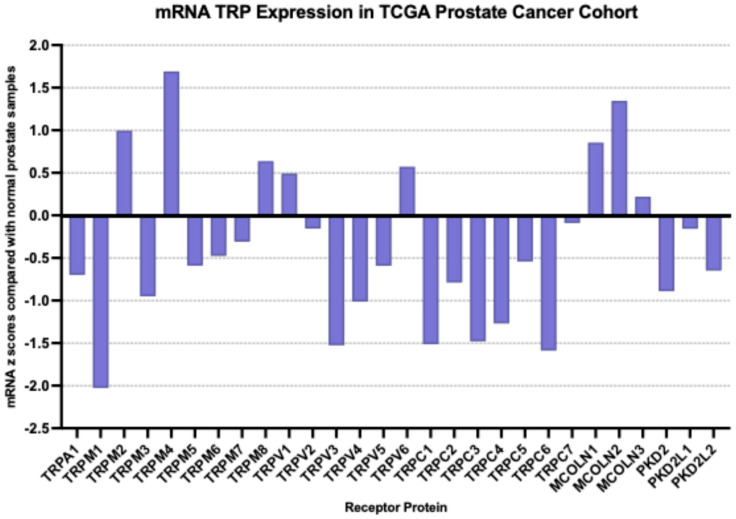
mRNA expression of TRP channels in prostate cancer (mRNA z scores compared with normal samples). Data are from the TCGA prostate cancer cohort.

At the mRNA level, the TRP channel that was most over-expressed in prostate cancer in the MSK cohort compared with a normal prostate was MCOLN2 (mean mRNA expression z score compared to normal samples = 2, SD = 2.01, *t* test compared with the second most expressed channel TRPV1 *p* = 0.0001, Figure 2); TRPM4, TRPM1, TRPM8 and MCOLN1 were also over-expressed, although at lower average levels. Most down-regulated mRNA levels were displayed by TRPV4, TRPC4, TRPC1 and TRPC6. MCOLN2 was also over-expressed in the TCGA prostate cancer cohort, although in somewhat lower levels (mean mRNA expression z score compared to normal samples = 1.34, SD = 1.2, Figure 3). In TCGA, TRPM4 displayed the highest expression with a mean mRNA expression z score compared to normal samples of 1.69 (*t* test compared with the second most expressed channel MCOLN2, *p* = 0.0001, Figure 3). The DKFZ study presented mRNA expression results only normalized as compared to all samples and therefore they were not directly comparable to the two other series which included comparisons normalized to samples with normal ploidy. TRPM4, together with the MCOLN2, MCOLN1, TRPC1 and TRPC6 the channels, had the best preserved expression.

At the protein level, the immunohistochemical evaluation by the human protein atlas disclosed moderate to high levels of expression for TRPC1 in all prostate cancer samples examined (n = 11, Figure 1). As mentioned above, TRPC1 was also robustly expressed in normal prostate, and this may explain the comparative down-regulation observed at the mRNA level in prostate cancer tissues. TRPML1, encoded by gene *MCOLN1*, and TRPML2, encoded by gene *MCOLN2*, were expressed at moderate to high levels in 80% and 90% of the prostate cancer samples examined. TRPM4, which was also well expressed in normal prostates, showed moderate to high expression in 75% of prostate cancers.

Prostate cancers with MCOLN2 over-expression (mean mRNA expression z score compared to normal samples > 2, n = 73) in the MSK prostate cohort were compared with prostate cancers with lower MCOLN2 mRNA expression (n = 77) from the same cohort (Table 1). The two groups showed no differences in their Gleason scores, aneuploidy prevalence or the presence of high TMB. Six samples (7.6%) had a TMB above 10 mutations/Mb (range 10.4 to 15.7 mutations/Mb). ERG fusions, affirmed either through array comparative genomic hybridization (aCGH) or gene expression, were significantly more prevalent in the group with MCOLN2 over-expression.

In the genomic series reported by the DKFZ (German Cancer Research Group), among 118 samples with mRNA expression data, 58 samples (49.2%) had a mean MCOLN2 mRNA expression z score compared to all samples above 0, and 60 samples (50.8%) had a mean MCOLN2 mRNA expression z score compared to all samples of 0 or below (Table 2). These series included early onset localized prostate cancer patients with a median age of 48 years old (range 32–52 years old) who had been treated with radical prostatectomy. The prevalence of high pre-operative Prostate-Specific Antigen (PSA) did not differ between the two groups with high and low MCOLN2 expression. High Gleason scores tended to be more frequent in the group with low MCOLN2 mRNA expression and this group had a significantly higher rate of biochemical recurrences than the group of patients with high MCOLN2 mRNA expression (Table 2). Similarly to the MSK series, ETS family transcription factor positivity was significantly more prevalent in the high MCOLN2 mRNA expression group. All samples of both groups had a TMB below 10 mutations/Mb.

The prostate cancer genomic study from TCGA had 494 patients with a mean age of 62 years old (Table 3). In this cohort, 166 patients (33.6%) had high MCOLN2 mRNA expression with a mean mRNA expression z score compared to normal samples above 2, and 328 patients (66.4%) had low MCOLN2 mRNA expression with a mean mRNA expression z score compared to normal samples of 2 or below. The two groups did not differ significantly in their mean age, their prevalence of high chromosome instability, as defined by the AS and the FGA score, or in their prevalence of high TMB (Table 3). Prostate cancers with high MCOLN2 mRNA expression were more frequently (69.9% of cases) positive for ERG fusions than cancers with low MCOLN2 mRNA expression (26.5% of cases positive, Fisher’s exact test *p* = 0.0001). Other mutations and copy number alterations were less frequent than ERG fusions in the prostate cancer TCGA cohort. *TP53* mutations encountered in 11.5% of the patients were equally distributed in cases with high and low MCOLN2 mRNA expression. The frequently encountered homodeletion of tumor suppressor *PTEN* was observed more often (23.2% of cases) in the group with high MCOLN2 mRNA expression than in the group with low MCOLN2 mRNA expression (14.5%, Fisher’s exact test *p* = 0.01).

The promoter of the *MCOLN2* gene did not possess clustered binding sites of ERG or AR and therefore the up-regulation of the MCOLN2 mRNA in ERG positive cases seems to be indirect (Table 4). The promoter possessed clustered binding sites for TCF4, SMAD family transcription factors and the EMT core transcription factor SNAIL2.

**Figure 4:**
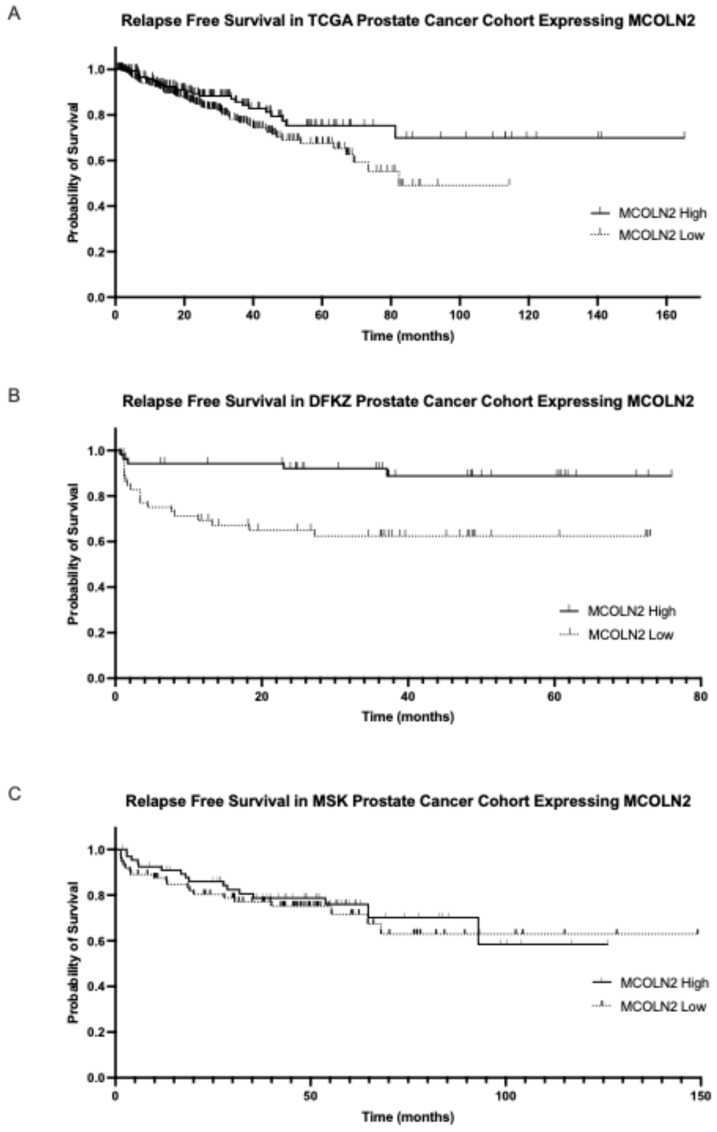
Biochemical relapse-free survival of the groups with high and low MCOLN2 mRNA expression. (**A**) TCGA cohort (Log Rank *p* = 0.05) and (**B**) DKFZ cohort (Log Rank *p* = 0.08). (**C**) Progression-free survival of prostate cancers with high and low mRNA MCOLN2 expression in the MSK cohort (Log Rank *p* = 0.6).

MCOLN2 mRNA expression had prognostic implications for biochemical relapse-free survival or disease-free survival in two of the three series examined. In the TCGA cohort, the group with high MCOLN2 mRNA expression had a longer biochemical relapse-free survival than the group with low MCOLN2 mRNA expression (Log Rank *p* = 0.05, Figure 4A). In the DKFZ cohort, the group with high MCOLN2 mRNA expression also had a trend towards longer biochemical relapse-free survival than the group with low MCOLN2 mRNA expression, although the difference did not attain statistical significance (Log Rank *p* = 0.08, Figure 4B). In contrast, the two groups did not differ in their disease-free survival in the MSK cohort (Log Rank *p* = 0.6, Figure 4C).

Regarding the other TRP channel with high expression in prostate cancer, TRPM4, prostate cancers with high mean mRNA expression in the MSK cohort, defined as a z score compared to normal samples above 1.5 (n = 42, 28%), did not differ from cancers with low TRPM4 mRNA expression in the presence of aneuploidy or the presence of ERG fusions, as measured by either aCGH (array Comparative Genomic Hybridization) or gene expression (Table 5). Although prostate cancers with lower Gleason scores of 6 or 7 were dominant in both groups, the group with high mean TRPM4 mRNA expression displayed a higher frequency of Gleason score 6 and 7 cancers (94.9%), while only 80% of cancers with low TRPM4 mRNA expression had Gleason scores of 6 or 7 (Fisher’s exact test *p* = 0.03, Table 4).

In the DKFZ cohort of early onset prostate cancers, the group with high mean TRPM4 mRNA expression, defined in this case as a z score above 0 compared to all samples (n = 64, 54.2%), also had a higher frequency of Gleason score 6 and 7 cancers (89.1%) compared to the group of lower TRPM4 expressors, who had Gleason scores of 6 and 7 in 79.6% of cases, but this difference did not reach statistical significance (Fisher’s exact test *p* = 0.2, Table 6). Prostate cancer patients with high mean TRPM4 mRNA expression more frequently had low pre-operative PSA values and a lower frequency of ETS family transcription factor positivity (Fisher’s exact test *p* = 0.02, Table 6).

Similarly, in the TCGA cohort, the group of patients with high mean TRPM4 mRNA expression (n = 295, 59.7%) had a significantly lower ERG positivity (31.5%) than the group with low mean TRPM4 mRNA expression (55.3%, Fisher’s exact test *p* = 0.0001, Table 7). The high mean TRPM4 mRNA expression group had a higher prevalence of chromosome instability, as measured by the FGA score but not the AS, compared to the low mean TRPM4 mRNA expression group of TCGA (Table 7).

The EPD database listed three promoters for *TRPM4* (TRPM4_1, TRPM4_2 and TRPM4_3). TRPM4_1 was active in the widest spectrum of human tissues and was the only promoter among the three that was active in prostate tissues and prostate cancer cell lines. TRPM4_1 possessed several clustered binding sites for SMADs transcription factors but no clusters for ERG, AR, TCF4 or SNAIL2 (Table 4).

**Figure 5:**
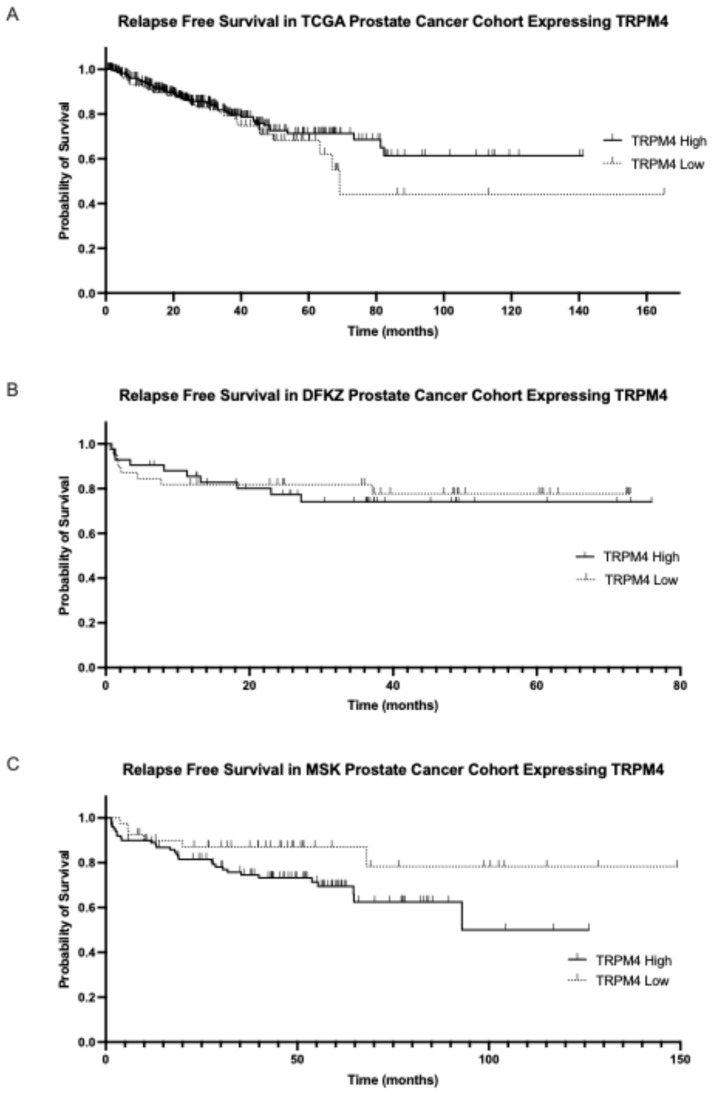
Biochemical relapse-free survival of the groups with high and low TRPM4 mRNA expression. (**A**) TCGA cohort (Log Rank *p* = 0.3) and (**B**) DKFZ cohort (Log Rank *p* = 0.8). (**C**) Progression-free survival of prostate cancers with high and low mRNA TRPM4 expression in the MSK cohort (Log Rank *p* = 0.08).

The prognosis of patients with high or low mean TRPM4 mRNA expression did not differ in any of the three cohorts examined (Figure 5) but a trend towards better survival of the group with low mean TRPM4 mRNA expression was observed in the MSK cohort (Log Rank *p* = 0.08, Figure 5C).

Table 8 summarizes the findings of the evaluations of MCOLN2 and TRPM4 mRNA expression in prostate cancer from the three genomic series examined.

## 3. Discussion

Calcium is an important second messenger involved in a plethora of cellular processes associated with cancer [17]. The regulation of cell proliferation and the cell cycle through the calcium calmodulin/calmodulin kinase axis is functional at mitosis entry and at the G1/S transition point [28]. Therefore, proteins and pathways regulating calcium concentrations in the cytoplasm may affect cancer development and progression if they become deregulated [27]. TRP channels represent a family of channels that facilitate the transport of cations across cell membranes. The six mammalian families of TRP channels have evolved to sense different physical and chemical conditions and stimuli and to orchestrate cellular responses through modulating anion concentrations [29]. Although their activation is coupled with membrane potential, TRP channels are expressed in both excitable and non-excitable cells, with the different members showing variable expression in different tissues. The permeability of channels for calcium is modulated by the presence of natural phytochemicals or temperature, translating these environmental conditions to calcium-mediated stimuli and the activation of intracellular signals. Other regulators of intracellular calcium, such as gap junctions, form an intricate network and may further propagate the effects of calcium cellular perturbation and connect it with cancer hallmarks, including epithelial–mesenchymal transition and stemness [29]. Gap junctions also participate in the communication of cancer cells with heterologous cells of the tumor micro-environment, connecting calcium currents and membrane electrical activity with the cancer stroma. The deregulation of TRP channels leads to organ dysfunction and contributes to a range of pathologic conditions [30]. In cancer, TRP channels influence proliferation, apoptosis and cell invasion [31,32]. Increased or decreased expressions of specific TRP channels compared to normal tissues have been reported in different cancers and have been linked with disease stage and prognosis [33,34,35,36]. In prostate cancer, TRP channels have also been implicated in cancer-associated processes, including cell proliferation and migration [31].

In the present study, we analyzed publicly available multiomic data to show that the expression of the TRP channel TRPML2, which is frequently over-expressed in prostate adenocarcinomas and encoded by the *MCOLN2* gene, is strongly associated with cases displaying ERG over-expression. ERG and other ETS family transcription factors are over-expressed in half of human prostate cancers as a result of fusions with the promoter sequence of the androgen receptor (AR)-dependent *TMPRSS2* gene [37,38]. *ERG* and *TMPRSS2* are both located in the chromosome arm 21q22, 3 Mb apart, and fusion events delete the coding sequence of *TMPRSS2*, juxtaposing its promoter with *ERG* and leading to ERG over-expression under the influence of AR. However, neither ERG nor AR possess clustered promoter binding sites in the promoter of the *MCOLN2* gene and, therefore, *MCOLN2* up-regulation in prostate cancers with ERG over-expression is not directly performed by these transcription factors. An indirect up-regulation through other transcription factors that are targets of ERG is possible. The *MCOLN2* promoter possesses clustered binding sites for transcription factor TCF4 of the WNT/β-catenin pathway, SMAD2/SMAD3 of the TGFβ pathway and the Epithelial–Mesenchymal Transition core transcription factor Slug (Snail2), which are regulated by ERG [39,40]. Despite the association with ERG fusions, prostate cancers with *MCOLN2* over-expression may have an improved prognosis, although this was not observed in all series. Variability in the prognosis based on mRNA expressions is not entirely unanticipated, given that some degree of instability of mRNA expressions can be expected, as the analysis represents a snapshot in time of mRNA expression for each sample. The variability of mRNA expression levels can be contrasted to alterations in DNA such as mutations and copy number changes that are more stable over time. The prognostic significance of *TMPRSS2: ERG* fusions in prostate cancer has been debated since the identification of this alteration in the disease [41]. A better prognosis of cases with specific sites of the fusion and resulting abundance of expression of the transcript has been reported [42]. The ratio of alternative transcripts has also been implicated in the prognostic significance of the fusion, as some transcripts act as negative regulators of others [43]. In addition to the presence of the fusion, the level of expression of ERG mRNA and protein may be critical for prognosis [44].

In contrast to our results that suggest that high MCOLN2 mRNA expression may be associated with better biochemical relapse-free survival outcomes, another study implied a worse overall survival of prostate cancers with high MCOLN2 mRNA [45]. However, this conclusion was based on an analysis of TCGA cohort, which contains only a few events.

The over-expression of another TRP channel, TRPM4, which is robustly expressed in prostate cancers, was inversely associated with ERG fusions or the over-expression of ERG and other ETS family transcription factors. The inverse association was statistically significant in two of the three series examined (DKFZ and TCGA). The promoter of TRPM4 that is functional in prostate tissues and prostate cancer did not possess clustered binding sites for ERG, AR, TCF4 or Slug but did have multiple binding sites for SMAD transcription factors, suggesting dominant regulation by the TGFβ pathway. Lower Gleason scores also tended to occur more often in cases with TRPM4 over-expression. Progression-free survival or biochemical relapse-free survival was not significantly different between the groups with high and low TRPM4 mRNA expression, although a trend for a worse disease-free survival of the group with higher TRPM4 mRNA expression was observed in the third series (MSK). These results suggest that the expression of TRPM4 channel is mostly favored in the sub-group of patients without ERG fusions and lower Gleason scores but has no confirmed effect on the prognosis of prostate cancer. ERG is a regulator of β-catenin and the WNT pathway and therefore low ERG expression may modulate WNT pathway outputs [38,46]. On the other hand, β-catenin is a regulator of the expression of gap junction proteins, i.e., connexins, and, therefore, it can affect calcium signaling and modulate the membrane potentials of cancer cells, further adding to potential deregulations effectuated by TRPM4 [29]. Moreover, TRPM4 levels were associated with an increased stability in β-catenin due to decreased phosphorylation and proteasome degradation [47]. The mechanism was traced to a TRPM4-dependent activation of the EGFR/AKT cascade, which leads to the inhibition of kinase GSK3β. Other receptor tyrosine kinases, such as Insulin-like Growth Factor Receptor (IGF1-R), are responsive to TRP channel-induced membrane depolarization and activate the AKT cascade [48]. This interconnected regulation exemplifies how developmental bioelectricity—where bioelectric signals direct cellular organization and fate through pathways like WNT—can be disrupted in cancer. In normal tissue development, bioelectric gradients and gap junction communication are essential for spatial patterning and morphogenesis, as they coordinate cellular responses to developmental signals [49]. However, in cancer, these bioelectric cues, influenced by channels like TRPM4, may become dysregulated, leading to aberrant cell proliferation and migration. Such bioelectric alterations underscore the need to understand TRPM4 and its interaction with the WNT/β-catenin and calcium signaling pathways, as these dynamics may offer insight into cancer progression and therapeutic targeting.

The varying expression of TRPs observed in this study highlights the importance of further research into both cell type-specific bioelectric signals and implicated ion channels [50]. Prognostic outcomes related to TRPM4 and TRPML2 expression between tissue types, as well as other ion channels, may be further modified by influences from the tumor micro-environment [51]. The TME can be influenced through chemical means but also by mechanical means such as increased tissue stiffness, interstitial fluid pressure and mechanical stress, enhancing malignancy [52,53]. During the development and growth of the tumor, mechanical stresses such as compression, tension and interstitial fluid pressure applied at areas of the membrane activate TRP channels, which translate these signals from physical to chemical [54]. TRPM4 channel, for example, activated by membrane stretch, transmits signals promoting cell proliferation and invasion [55,56]. Tissue-specific tumor micro-environment characteristics and the mechanical influences of prostate cancer, in addition to TRP channel over-expression, could account for the varying prognostic results and relapse-free survival rates observed across the three series examined in the study. For instance, compared to benign prostate tissue, prostate cancer has a distinctly denser extracellular matrix, contributing to higher rates of epithelial-to-mesenchymal transition (EMT) and increased metastasis [57,58,59].

Despite the lack of prognostic effect, TRPM4 has been shown previously to affect oncogenic processes [35,60]. In primary endometrial carcinoma cells, high TRPM4 mRNA expression correlated with the epithelial phenotype and less aggressive tumor behavior, consistent with the observed association with lower Gleason score prostate cancers in our study [35]. In contrast, the over-expression of TRP family members TRPV2 and TRPC1 in the same study of endometrial cancer cells was associated with the Epithelial–Mesenchymal Transition and more aggressive tumors. In prostate cancer, a small study that included 39 patients found an association of higher TRPM4 mRNA expression with higher Gleason scores [60]. Moreover, in contrast to the results in endometrial cancer, in vitro studies showed a decreased expression of EMT factor Snail1 and mesenchymal N-cadherin in prostate cancer cells after the knock-down of TRPM4 and up-regulation of epithelial E-cadherin. In colorectal cancer cells, tumor suppressor p53 was observed to suppress the transcription of TRPM4 and this suppression was associated with a shift of the cell cycle and accumulation of cells in the G1 phase [61]. These results imply that the role of TRPM4 in cancer processes is dependent on the specific cell micro-environment.

The targeting of TRP channels for therapeutic purposes in prostate cancer will require the discovery of clinical-grade inhibitors and the determination of specific sub-sets of patients and tumors that are sensitive to them, based on molecular characteristics and the specific roles that the respective targeted channels play in the pathophysiology of the disease [62]. Ion channels are targets for several classes of drugs and some drugs and natural chemicals that target TRP channels have anti-carcinogenic effects in cancer models [62]. For example, the natural compounds allicin and thymol have been reported to inhibit various cancer cells in vitro, although other mechanisms besides their interaction with TRP channels may mediate part of the anti-cancer effects [63,64]. Drug candidates for the inhibition of TRPM4 have been reported based on the optimization of the drug flufenamic acid and have been effective in inhibiting TRPM4 in vitro [65]. The study reported here provides a framework of the landscape of expression for two TRP channels that arise as potential candidates for targeting in prostate cancer based on their increased expression in the disease. As underlined in the discussion, besides the level of TRP channels expression, regulatory networks that integrate the ion fluxes and the downstream signaling will have to be considered in the therapeutic targeting of these receptors. The location of TRP channels in the cell and their interaction with partner proteins, which may also be expressed at varying levels in a given cancer and regulated by factors divergent from those regulating the TRP channels, would impart supplemental levels of complexity. In addition, the different biologic stages in the natural history of prostate cancer, which usually arise in the presence of an androgen ablation-sensitive disease, but develop castration resistance during their progression [66], will have to be considered in the efforts to target ion fluxes therapeutically.

## 4. Methods

The human protein atlas (www.proteinatlas.org (accessed on 18 January 2025)) is a resource which contains a compilation of protein and mRNA expressions from a variety of human tissues and corresponding cancers [67]. The atlas was used to examine the expression of TRP channels in normal prostate tissue and prostate cancer at the mRNA and protein level.

Three publicly available genomic studies with clinical and molecular data on prostate cancer patients were selected for the current analysis [3,4,5]. The first cohort published by investigators from the Memorial Sloan Kettering Cancer Center (MSK) included 150 mostly primary prostate cancer patients with data available for mRNA expression [3]. The Affymetrix Human Exon 1.0 platform was used for whole tumor mRNA expression analysis. The second prostate cancer cohort published from the German Cancer Research Center (DKFZ) included 118 patients with early onset prostate cancer [4]. The study used whole exome sequencing and analyzed whole tumor mRNA expression with the Illumina HiSeq 2000 flowcell protocol [4]. The prostate cancer cohort from TCGA, with 494 localized, mostly lymph node-negative prostate cancer patients, was the third cohort included in the analysis [5]. TCGA investigators used also whole exome sequencing for DNA analysis. RNA expression was normalized in TCGA with the RSEM (RNA-Seq by Expectation Maximization) algorithm and the results were presented as the Log RNA sequences in Reads per Kilobase Million (RPKM) [68].

The cBioCancer Genomics Portal (cBioportal, http://www.cbioportal.org, last accessed on 19 January 2025), a genomics site developed and maintained by MSKCC and other academic institutions, was used to interrogate sub-sets of cases with different levels of expression in TRP channels of interest in the three selected genomic cohorts [69,70]. For the analyses of parameters that were not directly provided in cBioportal, datasets were downloaded for additional calculations.

Sequences of gene promoters were identified in the Eukaryotic Promoter Database (EPD, https://epd.expasy.org/epd/, last accessed on 19 January 2025) [71]. The parameters used for motif search were as follows: −1000 to 100 base pairs from the transcription start site and a p cut-off value of 0.001. Binding motifs of transcription factors of interest were retrieved through the JASPAR CORE 2018 vertebrates database [72].

Statistical comparisons of categorical and continuous data were carried out with the Fisher exact test or the χ2 test and the *t* test. The Log Rank test was used to compare Kaplan–Meier survival curves. Survival analyses were performed using an online program (GraphPad Prism for graphing, https://www.graphpad.com/, last accessed on 17 January 2025). All statistical comparisons were considered significant if *p* < 0.05. Corrections for multiple comparisons were performed with the Benjamani–Hochberg FDR (false discovery rate) correction procedure.

## Figures and Tables

**Table 1:** Pathologic and genomic characteristics of prostate cancers with high or low mRNA expression of MCOLN2. Data are from the MSK prostate cohort. FGA: Fraction Genome Altered, TMB: Tumor Mutation Burden, NA: Not available.

	All Patients (n = 150) (%)	Patients with MCOLN2 > 2 (n = 73) (%)	Patients with MCOLN2 ≤ 2 (n = 77) (%)	*p*
Gleason score				
6–7	117 (84.2)	57 (86.4)	60 (82.2)	0.64
8–9	22 (15.8)	9 (13.6)	13 (17.8)	
NA	11	7	4	
Copy number cluster				
Flat	22 (14.7)	7 (9.6)	15 (19.5)	0.33 (diploid vs. aneuploid)
Hypodiploid	12 (8)	7 (9.6)	5 (6.5)	0.17 (χ^2^ all)
Diploid	34 (22.7)	14 (19.2)	20 (26)	
Hyperdiploid	82 (54.6)	45 (61.6)	37 (48)	
FGA				
<0.04	55 (43)	26 (39.4)	29 (46.8)	0.47 (<0.04 vs. >0.04)
0.04–0.24	63 (49.2)	36 (54.5)	27 (43.5)	0.42 (χ^2^ all)
>0.24	10 (7.8)	4 (6.1)	6 (9.7)	
NA	22	7	15	
ERG fusion by aCGH				
Positive	35 (23.3)	25 (34.2)	10 (13)	0.005
Negative	93 (62)	41 (56.2)	52 (67.5)	
Flat	22 (14.7)	7 (9.6)	15 (19.5)	
ERG fusion by gene expression				
Positive	74 (49.3)	47 (64.4)	27 (35.1)	0.0006
Negative	76 (50.7)	26 (35.6)	50 (64.9)	
TMB				
≤10/Mb	76 (92.7)	38 (92.7)	38 (92.7)	1
>10/Mb	6 (7.3)	3 (7.3)	3 (7.3)	
NA	68	32	36	

**Table 2:** Pathologic and genomic characteristics of prostate cancers with high or low mRNA expression of MCOLN2. Data are from the DKFZ early onset prostate cohort. TMB: Tumor Mutation Burden, NA: Not available.

	All Samples (n = 118) (%)	Samples with MCOLN2 > 0 (n = 58 Samples) (%)	Samples with MCOLN2 ≤ 0 (n = 60) (%)	*p*
Age				
Mean (SD)	47.2 (2.9)	47.3 (2)	47.1 (3.6)	0.7 (t), 0.54 (Mann–Whitney U)
Median (range)	48 (32–52)	48 (43–51)	48 (32–52)	
Gleason score				
6–7	100 (84.7)	53 (91.4)	47 (78.3)	0.07
8–10	18 (15.3)	5 (8.6)	13 (21.7)	
Pre-operative PSA				
≤20	86 (74.1)	43 (75.4)	43 (72.9)	0.83
>20	30 (25.9)	14 (24.6)	16 (27.1)	
NA	2	1	1	
Biochemical recurrence				
No	81 (77.1)	47 (90.4)	34 (64.2)	0.002
Yes	24 (22.9)	5 (9.6)	19 (35.8)	
NA	13	6	7	
ETS status				
Positive	86 (72.9)	49 (84.5)	37 (61.7)	0.007
Negative	32 (27.1)	9 (15.5)	23 (38.3)	
TMB				
≤10/Mb	114 (100)	56 (100)	58 (100)	
>10/Mb	0	0	0	
NA	4	2	2	

**Table 3:** Age and genomic characteristics of prostate cancers with high or low mRNA expression of MCOLN2. Data are from the TCGA prostate cohort. AS: Aneuploidy Score, FGA: Fraction Genome Altered, TMB: Tumor Mutation Burden, NA: Not available.

	All Patients (n = 494) (%)	Patients with MCOLN2 > 2 (n = 166) (%)	Patients with MCOLN2 ≤ 2 (n = 328) (%)	*p*
Age				
Mean (SD)	61 (6.8)	60.3 (6.9)	61.4(6.8)	0.09
Median (range)	61 (41–78)	61 (43–77)	62 (41–78)	
AS				
<4	368 (78.1)	134 (83.8)	234 (75.3)	
4–24	102 (21.7)	26 (16.2)	76 (24.4)	0.04
>24	1 (0.2)	0	1 (0.3)	0.11
NA	23	6	17	
FGA				
<0.04	181 (37)	57 (34.7)	124 (38.2)	0.48 (<0.04 vs. >0.04
0.04–0.24	267 (54.6)	98 (59.8)	169 (52)	0.13
>0.24	41 (8.4)	9 (5.5)	32 (9.8)	
NA	5	2	3	
ERG fusion				
Positive	203 (41.1)	116 (69.9)	87 (26.5)	0.0001
Negative	291 (58.9)	50 (30.1)	241 (73.5)	
TMB				
≤10/Mb	490 (99.2)	165 (99.4)	325 (99.1)	1
>10/Mb	4 (0.8)	1 (0.6)	3 (0.9)	

**Table 4:** Number of binding sites of key transcription factors in the promoters of TRP channel genes *MCOLN2* and *TRPM4*. Data are from the EPD database.

Transcription Factor	MCOLN2_1		TRPM4_1		TRPM4_2		TRPM4_3	
	Number of Motifs	Location Relative to TSS	Number of Motifs	Location Relative to TSS	Number of Motifs	Location Relative to TSS	Number of Motifs	Location Relative to TSS
ERG	1	−17	1	−15	3	−939, −433, −395	1	−417
AR	0		0		2	−939, −833	0	
DLX1	0		0		0		0	
FOXA1	0		0		0		1	−29
TP53	2	−857, −856	0		1	−780	0	
TCF4	5	−860, −795, −794, −632, −456	0		1	−753	7	−930, −654, −501, −269, −268, 38, 39
AP-1 (FOS:JUN)	1	−963	0		0		1	−978
SMAD2:SMAD3	5	−924, −813, −799, −701, 10	24	−984, −963, −941, −919 and 20 others	3	−421, −359, −181	5	−350, −287, −143, −119, 15
SMAD3	1	−461	1	−34	2	−603, −602	1	−400
SNAI2	4	−794, −632, −456, −379	1	−342	2	−753, −203	4	−654, −616, −600, −165

**Table 5:** Pathologic and genomic characteristics of prostate cancers with high or low mRNA expression of TRPM4. Data are from the MSK prostate cohort. FGA: Fraction Genome Altered, TMB: Tumor Mutation Burden, NA: Not available.

	All Patients (n = 150) (%)	Patients with TRPM4 > 1.5 (n = 42) (%)	Patients with TRPM4 ≤ 1.5 (n = 108) (%)	*p*
Gleason score				
6–7	117 (84.2)	37 (94.9)	80 (80)	0.03
8–9	22 (15.8)	2 (5.1)	20 (20)	
NA	11	3	8	
Copy number cluster				
Flat	22 (14.7)	7 (16.7)	15 (13.9)	0.66 (diploid versus aneuploid)
Hypodiploid	12 (8)	4 (9.5)	8 (7.4)	0.88
Diploid	34 (22.7)	8 (19)	26 (24.1)	
Hyperdiploid	82 (54.6)	23 (54.8)	59 (54.6)	
FGA				
<0.04	55 (43)	17 (48.6)	38 (40.9)	0.54 (<0.04 vs. >0.04)
0.04–0.24	63 (49.2)	16 (45.7)	47 (50.5)	0.68
>0.24	10 (7.8)	2 (5.7)	8 (8.6)	
NA	22	7	15	
ERG fusion by aCGH				
Positive	35 (23.3)	8 (19)	27 (25)	0.65
Negative	93 (62)	27 (64.3)	66 (61.1)	
Flat	22 (14.7)	7 (16.7)	15 (13.9)	
ERG fusion by gene expression				
Positive	74 (49.3)	16 (38.1)	58 (53.7)	0.1
Negative	76 (50.7)	26 (61.9)	50 (46.3)	
TMB				
≤10/Mb	76 (92.7)	23 (100)	53 (89.8)	0.17
>10/Mb	6 (7.3)	0	6 (10.2)	
NA	68	19	49	

**Table 6:** Pathologic and genomic characteristics of prostate cancers with high or low mRNA expression of TRPM4. Data are from the DKFZ early onset prostate cohort. TMB: Tumor Mutation Burden, NA: Not available.

	All Samples (n = 118) (%)	Samples with TRPM4 > 0 (n = 64 Samples) (%)	Samples with TRPM4 ≤ 0 (n = 54) (%)	*p*
Age				
Mean (SD)	47.2 (2.9)	47.4 (2.2)	47 (3.6)	0.46
Median (range)	48 (32–52)	48 (40–51)	47 (32–52)	
Gleason score				
6–7	100 (84.7)	57 (89.1)	43 (79.6)	0.2
8–10	18 (15.3)	7 (10.9)	11 (20.4)	
Pre-operative PSA				
≤20	86 (74.1)	52 (81.3)	34 (65.4)	0.058
>20	30 (25.9)	12 (18.7)	18 (34.6)	
NA	2	0	2	
Biochemical recurrence				
No	81 (77.1)	44 (73.3)	37 (82.2)	0.35
Yes	24 (22.9)	16 (26.7)	8 (17.8)	
NA	13	4	9	
ETS status				
Positive	86 (72.9)	41 (64.1)	45 (83.3)	0.02
Negative	32 (27.1)	23 (35.9)	9 (16.7)	
TMB				
≤10/Mb	114 (100)	62 (100)	52 (100)	
>10/Mb	0	0	0	
NA	4	2	2	

**Table 7:** Age and genomic characteristics of prostate cancers with high or low mRNA expression of TRPM4. Data are from the TCGA prostate cohort. AS: Aneuploidy Score, FGA: Fraction Genome Altered, TMB: Tumor Mutation Burden, NA: Not available.

	All Patients (n = 494) (%)	Patients with TRPM4 > 1.5 (n = 295) (%)	Patients with TRPM4 ≤ 1.5 (n = 199) (%)	*p*
Age				
Mean (SD)	61 (6.8)	60.8 (6.8)	61.4 (6.8)	0.33
Median (range)	61 (41–78)	61 (41–77)	62 (43–78)	
AS				
<4	368 (78.1)	224 (79.7)	144 (75.8)	
4–24	102 (21.7)	56 (19.9)	46 (24.2)	0.36
>24	1 (0.2)	1 (0.4)	0	
NA	23	14	9	
FGA				
<0.04	181 (37)	96 (33.1)	85 (42.7)	0.03
0.04–0.24	267 (54.6)	173 (59.7)	94 (47.2)	
>0.24	41 (8.4)	21 (7.2)	20 (10.1)	
NA	5	5	0	
ERG fusion				
Positive	203 (41.1)	93 (31.5)	110 (55.3)	0.0001
Negative	291 (58.9)	202 (68.5)	89 (44.7)	
TMB				
≤10/Mb	490 (99.2)	294 (99.7)	196 (99.1)	0.3
>10/Mb	4 (0.8)	1 (0.3)	3 (0.9)	

**Table 8:** Summary of the associations of TRP channels MCOLN2 and TRPM4 with prostate cancer clinical and genomic characteristics. TMB: Tumor Mutation Burden.

Characteristics in Prostate Cancer	MCOLN2	TRPM4
Mutations/amplifications	Both rare	Both rare
Over-expression	Over-expressed (highest TRP expression in MSK series, second-highest TRP expression in TCGA series).	Over-expressed (highest TRP expression in TCGA series, third-highest TRP expression in MSK series).
Gleason score	No clear association. Trend for high expression associated with low Gleason scores in one of the two series with data (DKFZ).	High expression associated with low Gleason scores (significant in one of the two series with data—MSK).
Ploidy	No association.	No association.
ERG fusions	Associated with high expression.	Associated with low expression (significant in 2 of the 3 series—DKFZ, TCGA).
TMB	High TMB very low in prostate cancer, not associated with MCOLN2 expression.	High TMB although very low in prostate cancer; not associated with TRPM4 expression.
Promoter regulation	Clusters of TCF4, TGFβ transducers and SNAI2 binding sites in promoter.	Clusters of TGFβ transducer binding sites in prostate cancer active promoter.
Prognosis	High expression associated with longer biochemical relapse-free survival (significant in one of the three series and a trend in another series).	No association with biochemical relapse-free survival.

## Data Availability

Data contained within the article.

## References

[1] Siegel R.L., Giaquinto A.N., Jemal A. (2024). Cancer statistics, 2024. CA Cancer J. Clin..

[2] Schaeffer E.M., Srinivas S., Adra N., An Y., Barocas D., Bitting R., Bryce A., Chapin B., Cheng H.H., D’Amico A.V. (2023). Prostate Cancer, Version 4.2023, NCCN Clinical Practice Guidelines in Oncology. J. Natl. Compr. Cancer Netw..

[3] Taylor B.S., Schultz N., Hieronymus H., Gopalan A., Xiao Y., Carver B.S., Arora V.K., Kaushik P., Cerami E., Reva B. (2010). Integrative genomic profiling of human prostate cancer. Cancer Cell.

[4] Gerhauser C., Favero F., Risch T., Simon R., Feuerbach L., Assenov Y., Heckmann D., Sidiropoulos N., Waszak S.M., Hübschmann D. (2018). Molecular Evolution of Early-Onset Prostate Cancer Identifies Molecular Risk Markers and Clinical Trajectories. Cancer Cell.

[5] Abeshouse A., Ahn J., Akbani R., Ally A., Amin S., Andry C.D., Annala M., Aprikian A., Armenia J., Arora A. (2015). The Molecular Taxonomy of Primary Prostate Cancer. Cell.

[6] Yang M., Brackenbury W.J. (2013). Membrane potential and cancer progression. Front. Physiol..

[7] Moreddu R. (2024). Nanotechnology and Cancer Bioelectricity: Bridging the Gap Between Biology and Translational Medicine. Adv. Sci..

[8] Balasubramanian S., Weston D.A., Levin M., Davidian D.C.C. (2024). Electroceuticals: Emerging applications beyond the nervous system and excitable tissues. Trends Pharmacol. Sci..

[9] Song M.Y., Yuan J.X. (2010). Introduction to TRP channels: Structure, function, and regulation. Adv. Exp. Med. Biol..

[10] Gees M., Colsoul B., Nilius B. (2010). The role of transient receptor potential cation channels in Ca^2+^ signaling. Cold Spring Harb. Perspect. Biol..

[11] Cervera J., Alcaraz A., Mafe S. (2014). Membrane potential bistability in nonexcitable cells as described by inward and outward voltage-gated ion channels. J. Phys. Chem. B.

[12] Humeau J., Bravo-San Pedro J.M., Vitale I., Nuñez L., Villalobos C., Kroemer G., Senovilla L. (2018). Calcium signaling and cell cycle: Progression or death. Cell Calcium.

[13] Levin M. (2014). Molecular bioelectricity: How endogenous voltage potentials control cell behavior and instruct pattern regulation in vivo. Mol. Biol. Cell.

[14] Sever R., Brugge J.S. (2015). Signal transduction in cancer. Cold Spring Harb. Perspect. Med..

[15] Jones C.A., Hazlehurst L.A. (2021). Role of Calcium Homeostasis in Modulating EMT in Cancer. Biomedicines.

[16] Bong A.H.L., Monteith G.R. (2018). Calcium signaling and the therapeutic targeting of cancer cells. Biochim. Biophys. Acta Mol. Cell Res..

[17] Marchi S., Giorgi C., Galluzzi L., Pinton P. (2020). Ca^2+^ Fluxes and Cancer. Mol. Cell.

[18] Barbonari S., D’Amore A., Palombi F., De Cesaris P., Parrington J., Riccioli A., Filippini A. (2022). Relevance of lysosomal Ca^2+^ signalling machinery in cancer. Cell Calcium.

[19] Garbincius J.F., Elrod J.W. (2022). Mitochondrial calcium exchange in physiology and disease. Physiol. Rev..

[20] Loncke J., Kaasik A., Bezprozvanny I., Parys J.B., Kerkhofs M., Bultynck G. (2021). Balancing ER-Mitochondrial Ca^2+^ Fluxes in Health and Disease. Trends Cell Biol..

[21] Umemura M., Nakakaji R., Ishikawa Y. (2023). Physiological functions of calcium signaling via Orai1 in cancer. J. Physiol. Sci..

[22] Amemiya Y., Maki M., Shibata H., Takahara T. (2023). New Insights into the Regulation of mTOR Signaling via Ca^2+^-Binding Proteins. Int. J. Mol. Sci..

[23] Marcelo K.L., Means A.R., York B. (2016). The Ca(2+)/Calmodulin/CaMKK2 Axis: Nature’s Metabolic CaMshaft. Trends Endocrinol. Metab..

[24] Nilius B., Owsianik G. (2011). The transient receptor potential family of ion channels. Genome Biol..

[25] Marini M., Titiz M., Souza Monteiro de Araújo D., Geppetti P., Nassini R., De Logu F. (2023). TRP Channels in Cancer: Signaling Mechanisms and Translational Approaches. Biomolecules.

[26] Gkika D., Prevarskaya N. (2011). TRP channels in prostate cancer: The good, the bad and the ugly?. Asian J. Androl..

[27] Litan A., Langhans S.A. (2015). Cancer as a channelopathy: Ion channels and pumps in tumor development and progression. Front. Cell Neurosci..

[28] Chafouleas J.G., Lagacé L., Bolton W.E., Boyd A.E., Means A.R. (1984). Changes in calmodulin and its mRNA accompany reentry of quiescent (G0) cells into the cell cycle. Cell.

[29] Aasen T., Leithe E., Graham S.V., Kameritsch P., Mayán M.D., Mesnil M., Pogoda K., Tabernero A. (2019). Connexins in cancer: Bridging the gap to the clinic. Oncogene.

[30] Yue L., Xu H. (2021). TRP channels in health and disease at a glance. J. Cell Sci..

[31] Déliot N., Constantin B. (2015). Plasma membrane calcium channels in cancer: Alterations and consequences for cell proliferation and migration. Biochim. Biophys. Acta.

[32] Dhaouadi N., Vitto V.A.M., Pinton P., Galluzzi L., Marchi S. (2023). Ca^2+^ signaling and cell death. Cell Calcium.

[33] Chelaru N.R., Chiosa A., Sorop A., Spiridon A., Cojocaru F., Domocos D., Cucu D., Popescu I., Dima S.O. (2022). The Association between TRP Channels Expression and Clinicopathological Characteristics of Patients with Pancreatic Adenocarcinoma. Int. J. Mol. Sci..

[34] Zhang C., Xu C., Ma C., Zhang Q., Bu S., Zhang D.L., Yu L., Wang H. (2022). TRPs in Ovarian Serous Cystadenocarcinoma: The Expression Patterns, Prognostic Roles, and Potential Therapeutic Targets. Front. Mol. Biosci..

[35] Van den Eynde C., De Clercq K., Van Bree R., Luyten K., Annibali D., Amant F., Han S., Van Nieuwenhuysen E., Baert T., Peeraer K. (2021). TRP channel expression correlates with the epithelial-mesenchymal transition and high-risk endometrial carcinoma. Cell Mol. Life Sci..

[36] Kiss F., Pohóczky K., Szállási A., Helyes Z. (2020). Transient Receptor Potential (TRP) Channels in Head-and-Neck Squamous Cell Carcinomas: Diagnostic, Prognostic, and Therapeutic Potentials. Int. J. Mol. Sci..

[37] Epstein R.J. (2021). The secret identities of TMPRSS2: Fertility factor, virus trafficker, inflammation moderator, prostate protector and tumor suppressor. Tumour Biol..

[38] Adamo P., Ladomery M.R. (2016). The oncogene ERG: A key factor in prostate cancer. Oncogene.

[39] Chakravarthi B.V.S.K., Chandrashekar D.S., Hodigere Balasubramanya S.A., Robinson A.D., Carskadon S., Rao U., Gordetsky J., Manne U., Netto G.J., Sudarshan S. (2018). Wnt receptor Frizzled 8 is a target of ERG in prostate cancer. Prostate.

[40] Ratz L., Laible M., Kacprzyk L.A., Wittig-Blaich S.M., Tolstov Y., Duensing S., Altevogt P., Klauck S.M., Sültmann H. (2017). TMPRSS2:ERG gene fusion variants induce TGF-β signaling and epithelial to mesenchymal transition in human prostate cancer cells. Oncotarget.

[41] Scaravilli M., Koivukoski S., Latonen L. (2021). Androgen-Driven Fusion Genes and Chimeric Transcripts in Prostate Cancer. Front. Cell Dev. Biol..

[42] Hermans K.G., Boormans J.L., Gasi D., van Leenders G.J., Jenster G., Verhagen P.C., Trapman J. (2009). Overexpression of prostate-specific TMPRSS2(exon 0)-ERG fusion transcripts corresponds with favorable prognosis of prostate cancer. Clin. Cancer Res..

[43] Hu Y., Dobi A., Sreenath T., Cook C., Tadase A.Y., Ravindranath L., Cullen J., Furusato B., Chen Y., Thangapazham R.L. (2008). Delineation of TMPRSS2-ERG splice variants in prostate cancer. Clin. Cancer Res..

[44] Font-Tello A., Juanpere N., de Muga S., Lorenzo M., Lorente J.A., Fumado L., Serrano L., Serrano S., Lloreta J., Hernández S. (2015). Association of ERG and TMPRSS2-ERG with grade, stage, and prognosis of prostate cancer is dependent on their expression levels. Prostate.

[45] Yu H., Xie M., Meng Z., Lo C.Y., Chan F.L., Jiang L., Meng X., Yao X. (2021). Endolysosomal ion channel MCOLN2 (Mucolipin-2) promotes prostate cancer progression via IL-1β/NF-κB pathway. Br. J. Cancer.

[46] Wu L., Zhao J.C., Kim J., Jin H.J., Wang C.Y., Yu J. (2013). ERG is a critical regulator of Wnt/LEF1 signaling in prostate cancer. Cancer Res..

[47] Sagredo A.I., Sagredo E.A., Cappelli C., Báez P., Andaur R.E., Blanco C., Tapia J.C., Echeverría C., Cerda O., Stutzin A. (2018). TRPM4 regulates Akt/GSK3-β activity and enhances β-catenin signaling and cell proliferation in prostate cancer cells. Mol. Oncol..

[48] Dai W., Bai Y., Hebda L., Zhong X., Liu J., Kao J., Duan C. (2014). Calcium deficiency-induced and TRP channel-regulated IGF1R-PI3K-Akt signaling regulates abnormal epithelial cell proliferation. Cell Death Differ..

[49] George L.F., Bates E.A. (2022). Mechanisms Underlying Influence of Bioelectricity in Development. Front. Cell Dev. Biol..

[50] Gorfe A.A. (2022). Biophysics of cancer. Biophys. J..

[51] Chen M.B., Javanmardi Y., Shahreza S., Serwinski B., Aref A., Djordjevic B., Moeendarbary E. (2023). Mechanobiology in oncology: Basic concepts and clinical prospects. Front. Cell Dev. Biol..

[52] Zhu P., Lu H., Wang M., Chen K., Chen Z., Yang L. (2023). Targeted mechanical forces enhance the effects of tumor immunotherapy by regulating immune cells in the tumor microenvironment. Cancer Biol. Med..

[53] Begum H.M., Shen K. (2023). Intracellular and microenvironmental regulation of mitochondrial membrane potential in cancer cells. WIREs Mech. Dis..

[54] Bera K., Kiepas A., Zhang Y., Sun S.X., Konstantopoulos K. (2022). The interplay between physical cues and mechanosensitive ion channels in cancer metastasis. Front. Cell Dev. Biol..

[55] Cáceres M., Ortiz L., Recabarren T., Romero A., Colombo A., Leiva-Salcedo E., Varela D., Rivas J., Silva I., Morales D. (2015). TRPM4 Is a Novel Component of the Adhesome Required for Focal Adhesion Disassembly, Migration and Contractility. PLoS ONE.

[56] Kappel S., Stokłosa P., Hauert B., Ross-Kaschitza D., Borgström A., Baur R., Galván J.A., Zlobec I., Peinelt C. (2019). TRPM4 is highly expressed in human colorectal tumor buds and contributes to proliferation, cell cycle, and invasion of colorectal cancer cells. Mol. Oncol..

[57] Li D., Xu W., Chang Y., Xiao Y., He Y., Ren S. (2023). Advances in landscape and related therapeutic targets of the prostate tumor microenvironment. Acta Biochim. Biophys. Sin..

[58] Lopez-Cavestany M., Hahn S.B., Hope J.M., Reckhorn N.T., Greenlee J.D., Schwager S.C., VanderBurgh J.A., Reinhart-King C.A., King M.R. (2023). Matrix stiffness induces epithelial-to-mesenchymal transition via Piezo1-regulated calcium flux in prostate cancer cells. iScience.

[59] Deng B., Zhao Z., Kong W., Han C., Shen X., Zhou C. (2022). Biological role of matrix stiffness in tumor growth and treatment. J. Transl. Med..

[60] Sagredo A.I., Sagredo E.A., Pola V., Echeverría C., Andaur R., Michea L., Stutzin A., Simon F., Marcelain K., Armisén R. (2019). TRPM4 channel is involved in regulating epithelial to mesenchymal transition, migration, and invasion of prostate cancer cell lines. J. Cell Physiol..

[61] Kappel S., Ross-Kaschitza D., Hauert B., Rother K., Peinelt C. (2022). p53 alters intracellular Ca^2+^ signaling through regulation of TRPM4. Cell Calcium.

[62] Kofman K., Levin M. (2024). Bioelectric pharmacology of cancer: A systematic review of ion channel drugs affecting the cancer phenotype. Prog. Biophys. Mol. Biol..

[63] Catanzaro E., Canistro D., Pellicioni V., Vivarelli F., Fimognari C. (2022). Anticancer potential of allicin: A review. Pharmacol. Res..

[64] De La Chapa J.J., Singha P.K., Lee D.R., Gonzales C.B. (2018). Thymol inhibits oral squamous cell carcinoma growth via mitochondria-mediated apoptosis. J. Oral. Pathol. Med..

[65] Ozhathil L.C., Delalande C., Bianchi B., Nemeth G., Kappel S., Thomet U., Ross-Kaschitza D., Simonin C., Rubin M., Gertsch J. (2018). Identification of potent and selective small molecule inhibitors of the cation channel TRPM4. Br. J. Pharmacol..

[66] Voutsadakis I.A., Papandreou C.N. (2012). The ubiquitin-proteasome system in prostate cancer and its transition to castration resistance. Urol. Oncol..

[67] Uhlén M., Fagerberg L., Hallström B.M., Lindskog C., Oksvold P., Mardinoglu A., Sivertsson Å., Kampf C., Sjöstedt E., Asplund A. (2015). Tissue-based map of the human proteome. Science.

[68] Li B., Dewey C.N. (2011). RSEM: Accurate transcript quantification from RNA-seq data with or without a reference genome. BMC Bioinform..

[69] Cerami E., Gao J., Dogrusoz U., Gross B.E., Sumer S.O., Aksoy B.A., Jacobsen A., Byrne C.J., Heuer M.L., Larsson E. (2012). The cBio cancer genomics portal: An open platform for exploring multidimensional cancer genomics data. Cancer Discov..

[70] Gao J., Aksoy B.A., Dogrusoz U., Dresdner G., Gross B., Sumer S.O., Sun Y., Jacobsen A., Sinha R., Larsson E. (2013). Integrative analysis of complex cancer genomics and clinical profiles using the cBioPortal. Sci. Signal.

[71] Meylan P., Dreos R., Ambrosini G., Groux R., Bucher P. (2020). EPD in 2020: Enhanced data visualization and extension to ncRNA promoters. Nucleic Acids Res..

[72] Khan A., Fornes O., Stigliani A., Gheorghe M., Castro-Mondragon J.A., van der Lee R., Bessy A., Chèneby J., Kulkarni S.R., Tan G. (2018). JASPAR 2018: Update of the open-access database of transcription factor binding profiles and its web framework. Nucleic Acids Res..

